# Supplementary material for: Interannual wave-driven shoreline change on the California coast
Source: Nat Commun. 2025 Nov 17;16:9967. doi: 10.1038/s41467-025-65944-0 (PMC12623795; doi:10.1038/s41467-025-65944-0)
Supplement: Supplementary file 1 — Supplementary Information [file 41467_2025_65944_MOESM1_ESM.pdf]

SUPPLEMENTARY INFORMATION

Interannual Wave-Driven Shoreline Change on the California Coast

William C. O'Reilly[a], Mark A. Merrifield[a], Laura Cagigal[b], Dayeon Yoon[a], Holden Leslie-Bole[a], Susheel Adusumilli[c], Adam P. Young[a], K. Vos[d], R. T. Guza[a]
[a] Scripps Institution of Oceanography, UC San Diego, La Jolla, CA, USA
[b] Universidad de Cantabria, Santander, Spain
[c] University of Oregon, Eugene, OR, USA
[d] OHB Digital Services, Konrad-Zuse-Str. 8, 28359 Bremen, Germany

Long-term shoreline trends and the PDO

The ERA5 wave reanalysis (1940-2023) is used to construct an 82-yr hindcast of the statewide average annual "wave-driven" mean shoreline change (Figure S1) based on the linear regression of 1985-2021 annual ERA5 offshore wave power against statewide averaged <CAcoastSat>, which yields slope of -0.61 (+/- 0.27) m $(m^3/s)^{-1}$. The hindcast shoreline position using wave ERA5 (black dashed line, Figure S1b) compares well with the Landsat shoreline (green dotted line), but underestimates 1984-1987 shoreline narrowing and 2012-2015 widening. Landsat shoreline imagery was relatively low spatial resolution and temporally sparse in the 1980's and 1990's, perhaps contributing to larger model errors.

The 82yr shoreline trends negative (black solid line, -0.054 ± 0.03 m/yr, Figure S1b) with a statewide average shoreline narrowing of ~ 4m since 1940, despite the more recent 1985-2021 period of mean shoreline position stability or slight widening derived from the Landsat images (green lines, S1b). Narrowing is more consistent with the perception of long-term shoreline erosion, particularly after the extensive pre-1960 artificial widening of CA beaches with harbor constructions[1,2]. This wave-driven estimate does not consider sand supply changes (river inputs, nourishments). Alternating multi-decadal time periods of modeled statewide narrowing and widening shoreline trends (positive and negative ERA5 wave power trends) align with warm (1977-1999) and cold (1947-1976, 2000-present) phases of the PDO (Figure S1). Longer-term shifts in atmospheric circulation and ocean heat content may also modulate wave power trends, influencing shoreline evolution beyond PDO-driven variability[3-7].

Following refs.[8,9] by parsing the hindcast into PDO cold and warm phases (blue and red shaded time periods, Figure S1), the power and shoreline trends are positive/negative in cold/warm phases (purple trends lines, Figure S1). The hindcast of the recent (2000-2021) cold phase trends compare favorably with the observed buoy (red solid line, Figure S1a) and <CAcoastSat> (green solid line, Figure S1b) trends. The PDO cold/warm phases are defined by the sign of the PDO index after applying a 5-yr running mean to the monthly PDO index to suppress ENSO modulation of the PDO signal[8-10].

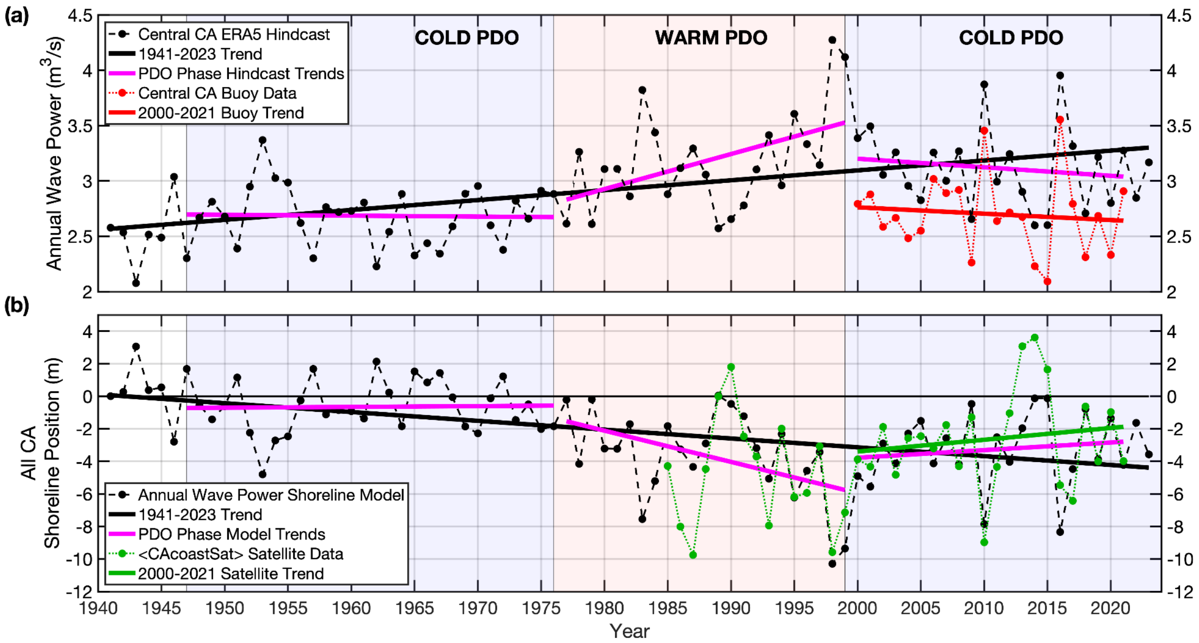

Figure S1. 1941-2023 wave-driven shoreline change hindcast. (a) ERA5 annual mean wave power (black dashed line) exhibits a positive long-term trend (black solid line, 0.009 m^3s^{-1}/yr). Multi-decadal wave power trends (purple lines) are negative/positive in cold/warm PDO phases. The ERA5 2000-2021 PDO cold phase power trend (right purple line) is consistent with the observed CA offshore trend (red solid line). (b) Estimated CA statewide average shoreline change since 1941 using ERA5 annual wave power and a linear regression of annual wave power against the <CAcoastSat> estimate of statewide annual relative shoreline position from 1985-2021 (green dotted line, vertically offset from 0 by the 1985-2021 mean of the modeled shoreline position for clarity). The 83-yr shoreline hindcast model (black dotted line) has a negative trend (black solid line, -0.054 m/yr) with a statewide average shoreline narrowing of ~4m since 1940. Model trends are positive/negative in cold/warm PDO phases (blue/red shaded time periods, purple lines). The ERA5 2000-2021 PDO cold phase shoreline trend (right purple line) is consistent with the observed <CAcoastSat> trend (green solid line). The Landsat estimates of more extreme annual beach narrowing (1985-1987) and recovery (2011-2013), associated with consecutive years of above or below average wave power, are underpredicted by an ERA5 reanalysis regression model (Figure S1). These errors are possibly an interannual cross-shore sand distribution analog to shoreline evolution model errors owing to missing seasonal bar dynamics[11] .

EOF Time-Longshore Plots

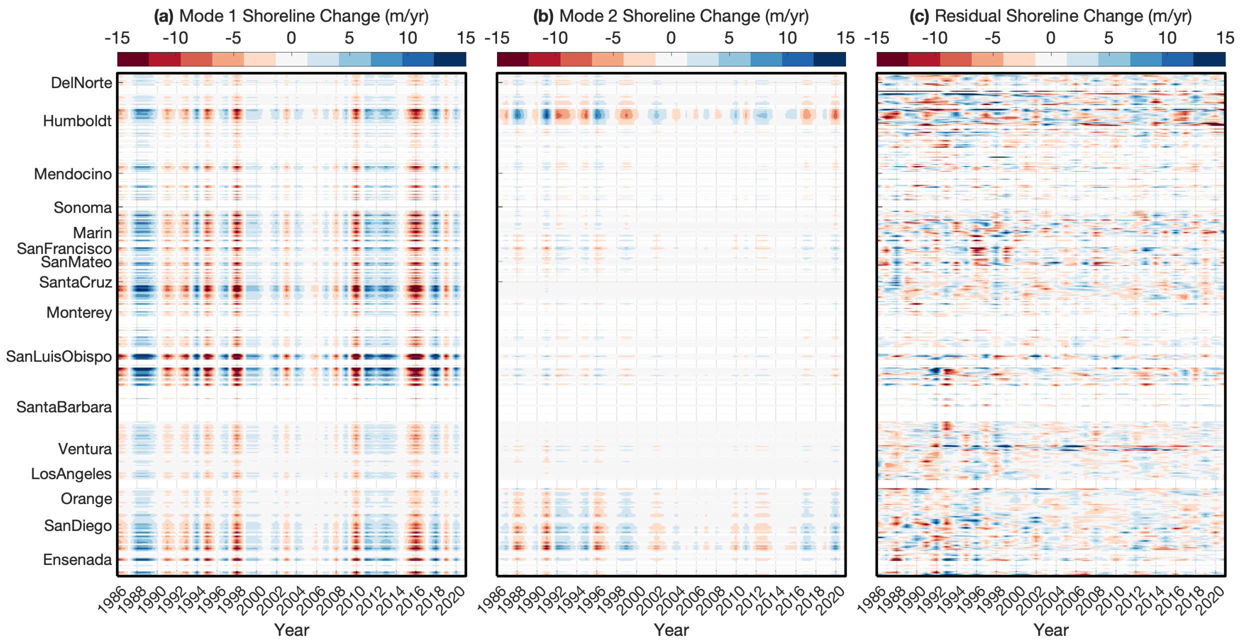

Figure S2. EOF analysis of <CAcoastSat> interannual shoreline change in Figure 4a. (a) Mode 1 (50% of variance) captures coherent statewide beach widening and narrowing (solid blue/red stripes, also see Figure 6a). (b) Mode 2 (7% of variance) shows regional-scale differences each year (alternating longshore blue/red widening/narrowing in the y dimension), primarily between Ensenada to Orange County in the south and Humboldt-Del Norte County in the north (also see Figure 6b). (c) The remaining mode 1+2 residual change (43% of variance) is more localized change. The statewide means for each year are only significantly greater than 0 for mode 1 (see Figure 6c-e).

Results Using Most Recent CoastSat v1.6 Data (June 7, 2025)

CoastSat data version 1.2 (https://zenodo.org/records/15614554), is used in the main paper. It is the same CoastSat version used in the previous study of ENSO teleconnections to both beach narrowing and widening throughout the Pacific Basin.[7] Updated versions of the CoastSat data are available from the same zendo.org source that both extend the time series beyond 2021 and include improvements in georeferencing, tidal corrections, and QA/QC. While these changes are significant for specific time periods and shoreline locations, the impact on the (highly time-space averaged) annual-regional results presented here are small. Figures S3,S4 are reproductions of Figures 4,5 using CoastSat v1.6 data instead of 1.2. The correlation between annual CA-wide shoreline change and ERA5 offshore wave power change improves slightly but not significantly (r^2 = 0.72 Figure 4b; r^2 =0.74 Figure S3b). The most notable difference is a mild reduction in EOF mode 2 variance in the early Landsat years in S. CA (1985-1994, Figures 5f,S4f). Nevertheless, 1988 and 1991 continue to stand out in mode 2 as years with significant differences in S. and N. CA shoreline change behavior.

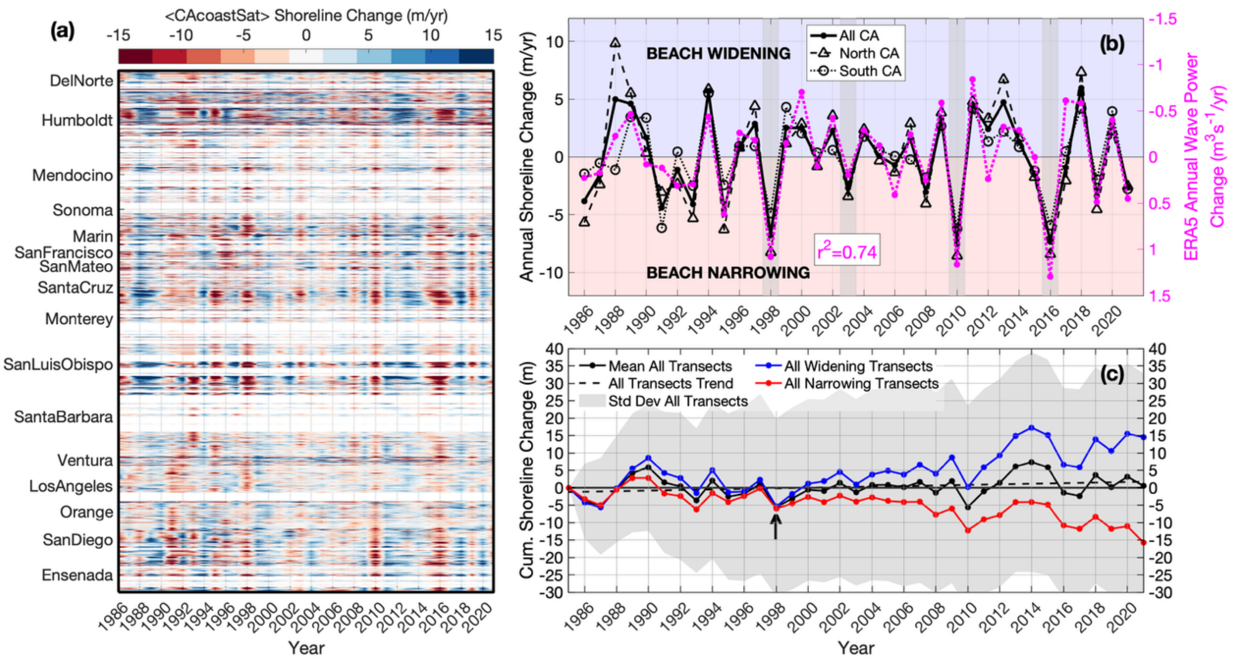

Figure S3. Reproduction of Figure 4 using CoastSat v1.6 data. Panel a,b changes are very subtle compared to Figure 4a,b. Correlation between statewide mean change and ERA5 wave power change (panel b, $r^2 = 0.74$) is slightly improved. The all transect 1985-2021 trend is reduced from a small positive trend in Figure 4c to near 0 (panel c, dashed black line).

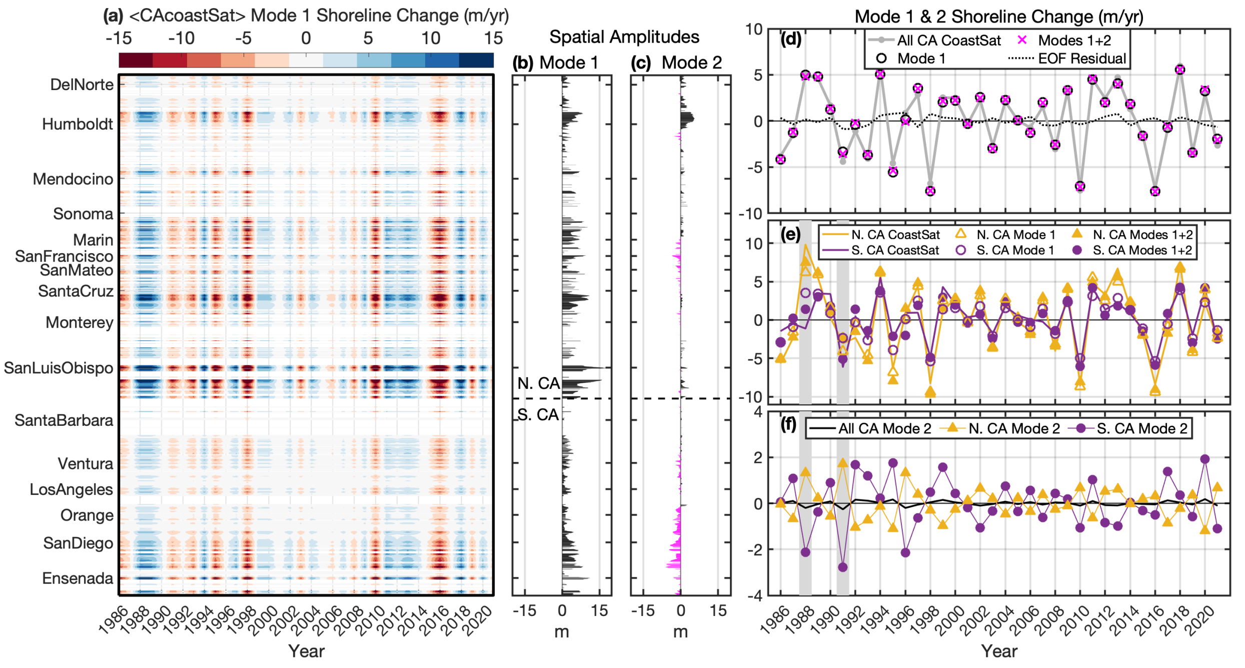

Figure S4. Reproduction of (a) Figure S2a and (b-f) Figure 5a-e using CoastSat v1.6 data. Most noticeable change is the reduced S. CA mode 2 amplitude outliers (panel f, for 1988 and 1991 shaded years) compared to Figure 5e.

Additional CoastSat Validation Results

A global mean of the five San Diego beach <CAcoastSat> and <Survey MHW> annual shoreline position time series from Figure 2b (Figure S5a) has high overall correlation (r^2 =0.89) and low RMSE (2.3m) relative to the 20yr net change of ~10m. The shortest timescale interannual shoreline change, the difference between sequential annual means, is correlated with wave power change (Figure S5b). <CAcoastSat> change is more highly correlated with wave power change (r^2 = 0.76) than <Survey MHW> (r^2 = 0.47).

Assuming <Survey MHW> change as groundtruth, the <CAcoastSat> change error is also significantly correlated with wave power (r^2 =0.62, Figure S5c). This is consistent with a (not corrected for) annual mean runup anomaly, driven by the offshore wave power anomaly, effectively amplifying the yearly <CAcoastSat> shoreline change signal, but with low bias and little cumulative impact on longer timescale skill (the high correlation and low RMSE in Figure S5a).

Alternatively, prior to the mid-2017 introduction of truck LiDAR, the majority of the beach surveys were done quarterly (either airborne LiDAR or ATV GPS), which may dampen the variance of the resulting annual means compared to the satellite estimates. Extension of the validation time series with future truck LiDAR surveys should provide more insight.

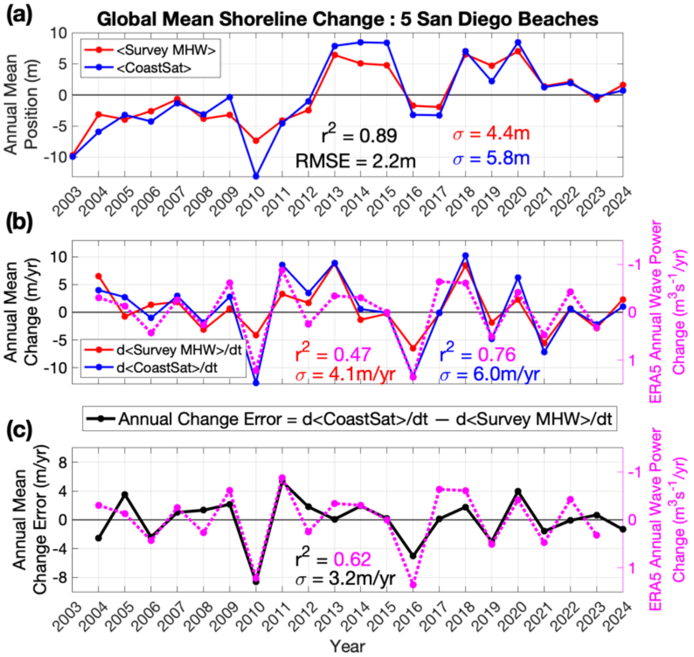

Figure S5. Time series of offshore ERA5 wave power, <Survey MHW> and <CAcoastSat> shoreline change. (a) Global annual mean shoreline positions averaged over 5 San Diego beaches (Figure 2b). (b) Year-to-year position change is inversely correlated with offshore wave power change (purple line, right axis, note reversed y-scale). (c) <CAcoastSat> change error

relative to <Survey MHW> position change is also inversely correlated with offshore wave power, consistent with a (not corrected for) wave power-driven annual mean runup anomaly.

References

1. Flick, R. E. The myth and reality of southern California beaches, *Shore and Beach*, **61** (3), 3-13 (1993).
2. Patsch, K., & Griggs, G. B. Littoral cells, sand budgets, and beaches: Understanding California's shoreline. *Institute of Marine Sciences, University of California, Santa Cruz* (2006). https://www.coastal.ca.gov/coastalvoices/resources/2006-LittoralCells.pdf
3. Trenberth KE, et al. Observations: Surface and Atmospheric Climate Change. In Climate Change 2007: The Physical Science Basis. Contribution of Working Group I to the Fourth Assessment Report of the Intergovernmental Panel on Climate Change. (2007).
4. Gemmrich, J., Thomas, B., & Bouchard, R. Observational changes and trends in northeast Pacific wave records. *Geophys. Res. Lett.*, **38**(22), L22601 (2011). https://doi.org/10.1029/2011GL049518
5. Reguero, B. G., Losada, I. J., & Mendez, F. J. A recent increase in global wave power as a consequence of oceanic warming. *Nat. Comm.,* **10**(1), 205 (2019). https://doi.org/10.1038/s41467-018-08066-0
6. Stopa, J. E., Ardhuin, F., Stutzmann, E., & Lecocq, T. Sea state trends and variability: Consistency between models, altimeters, buoys, and seismic data (1979-2016). *J. Geophys. Res.: Oceans*, **124**(6), 3923–3940 (2019). https://doi.org/10.1029/2018JC014607
7. Timmermans, B. W., Gommenginger, C. P., Dodet, G., & Bidlot, J.-R. Global wave height trends and variability from new multimission satellite altimeter products, reanalyses, and wave buoys. *Geophys. Res. Lett.,* **47**(9), e2019GL086880 (2020). https://doi.org/10.1029/2019gl086880
8. Wang, X. L., & Swail, V. R. Changes of extreme wave heights in northern hemisphere oceans and related atmospheric circulation regimes. *J. Clim.*, **14**(10), 2204–2221 (2001). https://doi.org/10.1175/1520-0442(2001)014%3C2204:COEWHI%3E2.0.CO;2
9. Bromirski, P. D., Cayan, D. R., Helly, J., & Wittmann, P. Wave power variability and trends across the North Pacific. *J. Geophys. Res. Oceans,* **118**, 6329–6348 (2013). doi:10.1002/2013JC009189
10. Mantua, N. J., Hare, S. R., Zhang, Y., Wallace, J. M., and Francis, R. C. A Pacific interdecadal climate oscillation with impacts on salmon production. *Bull. Amer. Meteor. Soc.*, **78**, 1069–1080 (1997). https://doi.org/10.1175/15200477(1997)078<1069:APICOW>2.0.CO;2.
11. Ferreira, A. M., Coelho C., & Silva, P. A. Numerical evaluation of the impact of sandbars on cross-shore sediment transport and shoreline evolution, *J. Environ. Manag.*, **370** (2024). https://doi.org/10.1016/j.jenvman.2024.122835
